# Supplementary material for: Maximizing Sensory Dynamic Range by Tuning the Cortical State to Criticality
Source: PLoS Comput Biol. 2015 Dec 1;11(12):e1004576. doi: 10.1371/journal.pcbi.1004576 (PMC4666488; doi:10.1371/journal.pcbi.1004576)

Figure S1. Continuum of cortical states is robust to variations in the analysis parameters TH and DT. Shown are all of the avalanche size distributions (with log-log axes), vertically shifted and color coded according to their κ values (high and red for high κ; low and blue for low κ). A subset of these distributions is shown in Fig 2d of the main manuscript. Each panel shows distributions created with different parameters TH and DT. We tested this for TH= 5, 10, and 20 spikes (left to right) and DT= 5, 10, and 20 ms (top to bottom). Robust to these parameter changes is the general observation of a continuum of cortical states ranging from weakly correlated small avalanches (blue) to strongly correlated large avalanches (red). As DT increases (top to bottom), κ systematically tends toward higher values. Changing TH (left to right) also can change κ, but without a clear systematic trend. The parameter DT defines the duration of the time bins used to make the spike count time series. The parameter TH is the threshold for defining avalanches. (See Materials and Methods for more description of these parameters.)

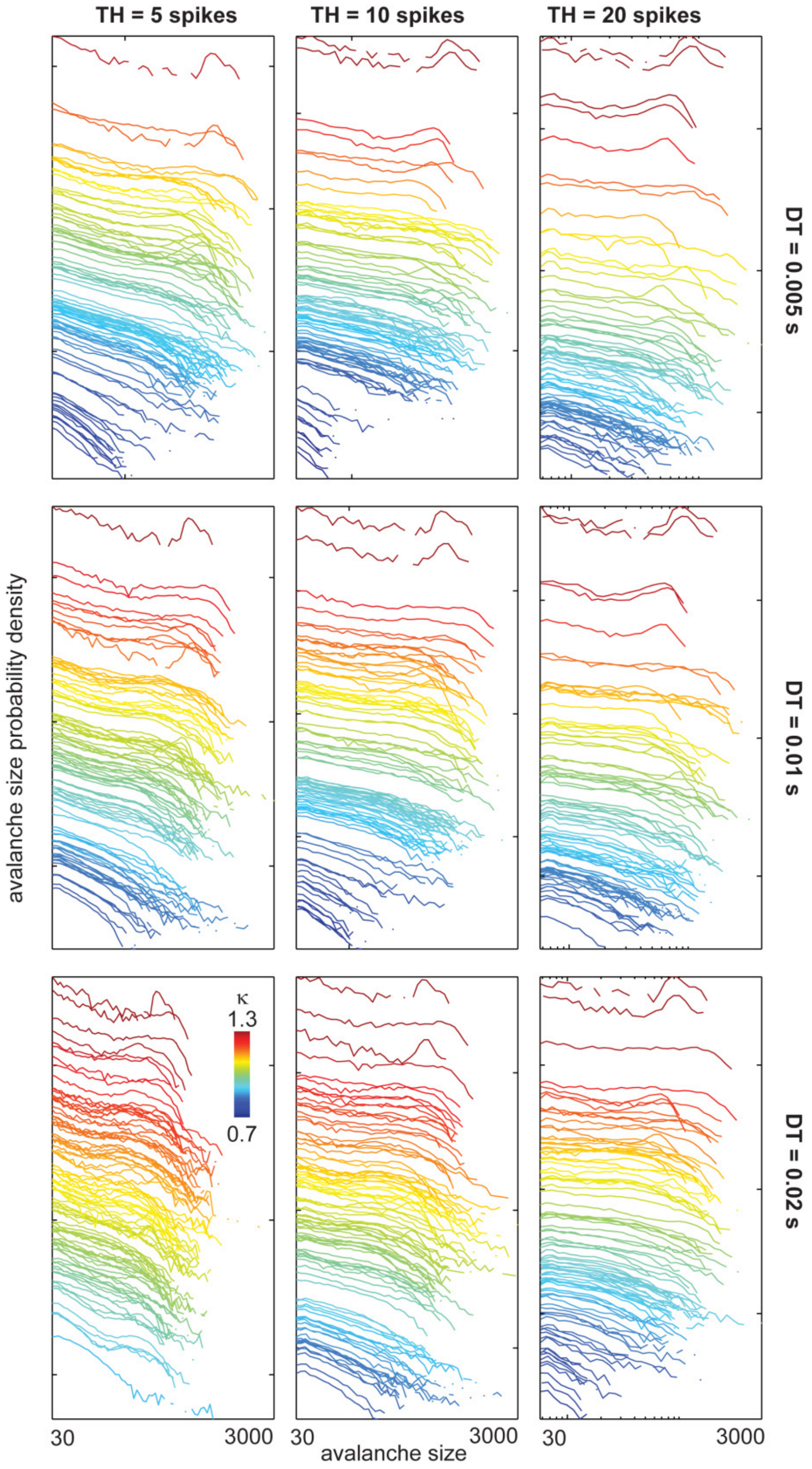

Supplement: S1 Fig — Shown are all of the avalanche size distributions (with log-log axes), vertically shifted and color coded according to their κ values (high and red for high κ; low and blue for low κ). A subset of these distributions is shown in Fig 2D of the main manuscript. Each panel shows distributions created with different parameters TH and DT. We tested this for TH = 5, 10, and 20 spikes (left to right) and DT = 5, 10, and 20 ms (top to bottom). Robust to these parameter changes is the general observation of a continuum of cortical states ranging from weakly correlated small avalanches (blue) to strongly correlated large avalanches (red). As DT increases (top to bottom), κ systematically tends toward higher values. Changing TH (left to right) also can change κ, but without a clear systematic trend. The parameter DT defines the duration of the time bins used to make the spike count time series. The parameter TH is the threshold for defining avalanches. (See Materials and Methods for more description of these parameters.) (PDF) [file pcbi.1004576.s001.pdf]
