# Supplementary material for: Maximizing Sensory Dynamic Range by Tuning the Cortical State to Criticality
Source: PLoS Comput Biol. 2015 Dec 1;11(12):e1004576. doi: 10.1371/journal.pcbi.1004576 (PMC4666488; doi:10.1371/journal.pcbi.1004576)
Supplement: S2 Fig — Shown are summary plots of dynamic range ∆ versus deviation from power-law κ. These are comparable to the data shown in Fig 4D of the main manuscript. Since κ depends on the analysis parameters DT and TH, as shown in S1 Fig, the ∆ versus κ relationship could also depend on these parameters. We tested this for TH = 5, 10, and 20 spikes (left to right) and DT = 5, 10, and 20 ms (top to bottom). The central panel is closest to the parameter values used for the analysis shown in Fig 4D. Most of these parameter combinations show a clear peak in dynamic range near κ = 1. Thus, we conclude that our primary finding is largely robust to these changes in analysis parameters. (PDF) [file pcbi.1004576.s002.pdf]

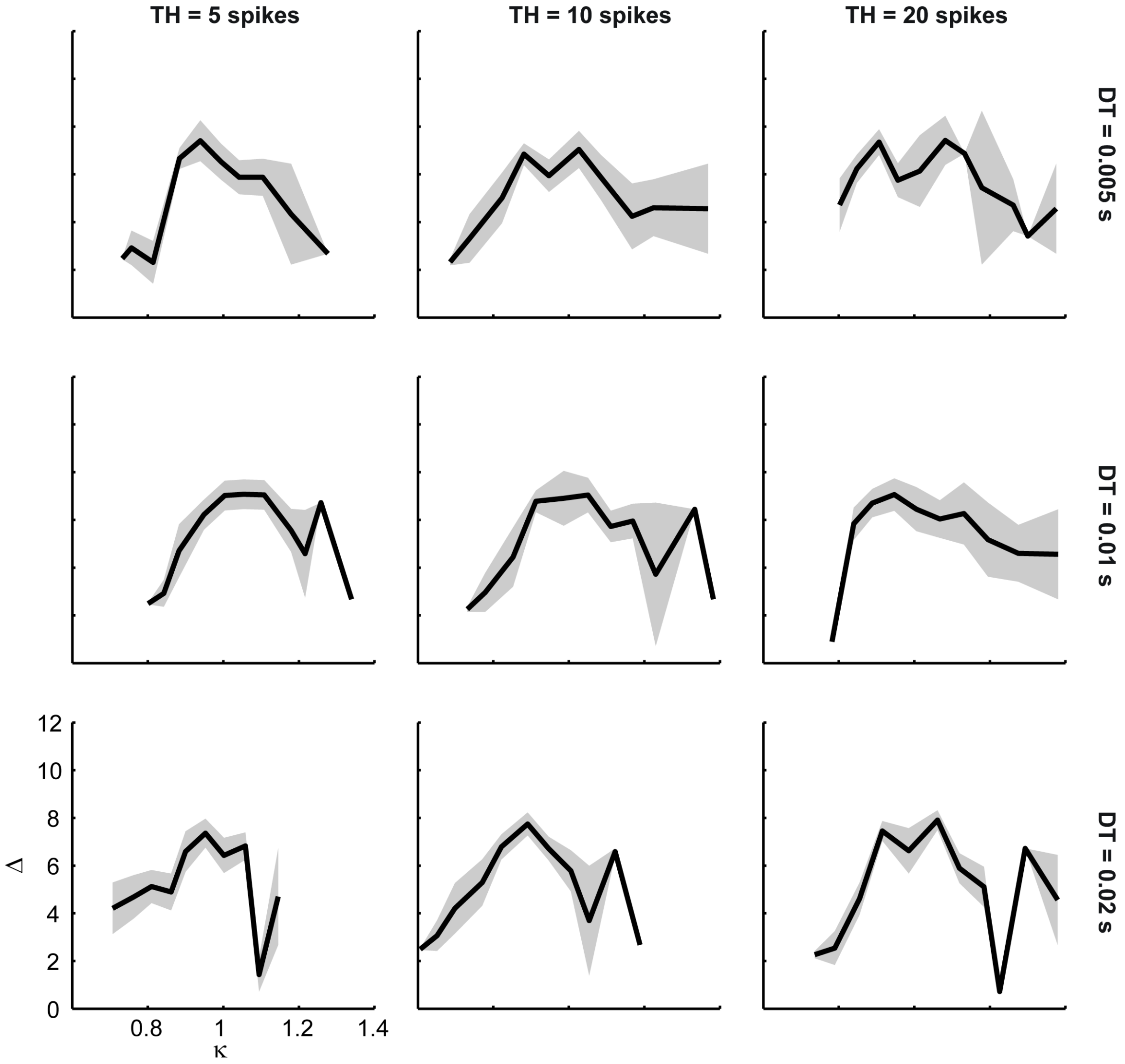

Figure S2. Peak dynamic range near κ=1 is robust to variations in the analysis parameters TH and DT. Shown are summary plots of dynamic range Δ versus deviation from power-law κ. These are comparable to the data shown in Fig 4d of the main manuscript. Since κ depends on the analysis parameters DT and TH, as shown in Supplementary Fig S1, the Δ versus κ relationship could also depend on these parameters. We tested this for TH= 5, 10, and 20 spikes (left to right) and DT= 5, 10, and 20 ms (top to bottom). The central panel is closest to the parameter values used for the analysis shown in Fig 4d. Most of these parameter combinations show a clear peak in dynamic range near κ=1. Thus, we conclude that our primary finding is largely robust to these changes in analysis parameters.
